# Vitamin D and Albumin Deficiency in a Swiss Orthopaedic Surgery In-Patient Cohort

**DOI:** 10.3390/jcm13092577

**Published:** 2024-04-27

**Authors:** Craig David Kingston, Simone Santini, Dorian Hauke, Victor Valderrabano

**Affiliations:** 1 Faculty of Medicine, University of Basel, 4001 Basel, Switzerland; c.kingston@unibas.ch; 2 Swiss Ortho Center, Swiss Medical Network, Schmerzklinik Basel, Hirschgässlein 15, 4010 Basel, Switzerland; s.santini@unicampus.it (S.S.); dhauke@swissmedical.net (D.H.); 3 Department of Orthopaedic and Trauma Surgery, Fondazione Policlinico Campus Bio-Medico, Via Alvaro del Portillo, 00128 Rome, Italy; 4 Research Unit of Orthopaedic and Trauma Surgery, Università Campus Bio-Medico di Roma, Via Alvaro del Portillo, 00128 Rome, Italy

**Keywords:** vitamin D, vitamin D deficiency, albumin deficiency, orthopaedics, rehabilitation

## Abstract

**Background:** Vitamin D and protein deficiencies are common conditions in the general population. In Orthopaedic surgery, they can result in wound complications or poor bone healing. The goal of this study was, therefore, to determine the prevalence of vitamin D and albumin deficiencies in patients scheduled for elective Orthopaedic procedures. **Methods:** We performed an observational, noninterventional study using the demographic characteristics via means chart analysis of in-patients with an elective surgery in a single Swiss Orthopaedic centre. The following variables were collected and analysed: age, gender, BMI, ASA score, rate of vitamin D supplementation before surgery, and serum preoperative levels of vitamin D, albumin, Haemoglobin, calcium, and phosphate. **Results:** A total of 336 patients were analysed; there were 218 women (64.9%) and 118 men (35.1%). The average age was 59.4 years (17–89 years). The average BMI was 26.8 kg/m^2^ (17.8–37.6) and the average ASA score was II (I–III). The overall prevalence of vitamin D deficiency was 82.1%, being more common in the male (89.8%) than female patients (77.9%). Patients who received vitamin D supplements before surgery had an average vitamin D level in the normal range. Of the subgroup of 170 patients who were over 60 years of age, 78.8% of the patients were Vitamin D deficient, with deficiency once again being more common in men (93.3%) than in women (92%). Albumin deficiency was documented in 58.9% of the cases. A total of 62.8% of all the female patients were albumin deficient, and 51.7% of all male patients were. Of the subgroup of 170 patients who were over 60 years of age, 71.8% of the patients were albumin deficient, with the deficiency being practically identical in men (71.1%) and women (72%). **Conclusions:** Despite increased awareness in the medical community, vitamin D and albumin deficiencies remain highly prevalent in elective Orthopaedic patients. Pre/perioperative screening and addressing possible vitamin D and albumin deficiencies are of great importance for good outcomes in Orthopaedic surgery.

## 1. Introduction

Vitamin D deficiency, as well as dietary protein deficiency, are present within the general population and are factors that can lead to delayed healing after Orthopaedic operations [1,2,3]. Approximately 50% of all Swiss adults are vitamin D deficient. Vitamin D deficiency is particularly prevalent among adults in northern Europe, and the Swiss health ministry recommends supplementation for all adults over 60 [1]. According to the last population-based study from 2012, variations can be seen within the Swiss population, depending on factors like time of year (higher levels in summer, lower levels in winter) and region (the highest levels being seen in the Italian-speaking parts of the country, the lowest levels in the French-speaking areas, and the German-speaking patients ranked second). The language-based differences remain even when cofounders are taken into consideration [4].

Despite the recommendations, many factors (including poor adherence, obesity, or malabsorption) can lead to persisting vitamin D deficiency, even with supplementation [2]. Hypovitaminosis D is associated with cardiovascular disease, metabolic syndrome, type 2 diabetes mellitus, and cancer, as well as with increased mortality in the general population. Further, vitamin D deficiency is related to depression and impaired cognitive function [5]. Vitamin D deficiency in the perioperative period has been associated with increased risk of postoperative infections, longer rehabilitation times, delayed healing or non-healing after Orthopaedic surgeries (such as nonunion of arthrodeses or osteotomies), increased pain scores and, to some extent, even contribute to higher mortality rates [6].

Epidemiological data for average serum albumin levels are not available. Diagnosed cases of protein energy malnutrition (for which serum albumin is considered to be a reliable marker) remain relatively low in countries with a high sociodemographic index (approximately 923/100,000 inhabitants in 2019) [7]. However, it is believed that these numbers may be underestimated. In addition, dietary protein deficiency is underdiagnosed, particularly in elderly patients, which can lead to an increased fracture risk or poor wound and tissue healing following surgery [8]. The measurement of the stores of vitamin D is generally performed via serological measurement of the level of 25OHD, as this is a more accurate reflection of the vitamin D reserves than measurement of the active metabolite 1,25(OH)2D [9].

No up-to-date data for these two issues (vitamin D insufficiency and hypoproteinaemia) exist for Swiss elective Orthopaedic surgery patients. The primary goal of this observational, noninterventional investigation was to determine the prevalence of vitamin D and albumin deficiencies in patients scheduled for elective Orthopaedic procedures.

## 2. Materials and Methods

A retrospective chart analysis of the Orthopaedic in-patient records from one calendar year (1 January 2022–31 December 2022) was performed. All the patients underwent elective surgery in a single Orthopaedic centre by the senior author. The data regarding gender, weight, height, BMI, ASA classification, whether vitamin D supplementation was prescribed prior to surgery, and the preoperative levels of serum vitamin D, serum albumin, Haemoglobin, calcium, and phosphate were collected and analysed. The cutoff levels for deficiencies were 75 nmol/L for vitamin D, 35.0 g/L for albumin, 120 g/L for Haemoglobin, 2.1 mmol/L for calcium, and 0.81 mmol/L for phosphate.

To be included in this study, the patients must have attended the above-named clinic, planned for elective surgery, and undergone the necessary perioperative serological investigations and the planned Orthopaedic intervention. The presence of a documented refusal was considered to be the only exclusion criteria. Ethical approval was sought and obtained from the local IRB institution.

## 3. Results

### 3.1. Data

A total of 336 patients were included in the analysis (Table 1). The group was predominantly female (n = 218, 64.9%). The average age was 59.4 years. Most patients were in good health, with an average ASA score of II and a maximum of III. A deficient level of vitamin D was detected preoperatively in 276 patients (82.1%) (Table 2). An albumin deficiency was detected in 198 cases (58.9%) (Table 2). Almost half of the patients had preoperative anaemia (n = 141, 41.9%), with a cutoff of 120 g/L. Of the included patients, 30% had deficient levels of calcium and 30.1% had deficient levels of phosphate.

### 3.2. Vitamin D Analysis

In both the women and men who underwent surgery, there was a high frequency of vitamin D deficiency (Table 3). A total of 77.9% of the women and 89.8% of the men were vitamin D deficient, with a cutoff of 75 nmol/L. The average level for women in this study was 57.7 nmol/L, and for men it was 53 nmol/L. In the group with a deficiency, the average was 49.1 nmol/L for men and 46.4 nmol/L for women. Only 18 (5.3%) patients were receiving vitamin D supplementation, with an average level of 86.9 nmol/L (range 62–163 nmol/L). Among the 210 patients (62.5% of the total group) with a raised BMI (>25 kg/m^2^), the average vitamin D level was 55.6 nmol/L, in comparison to the entire group at 56.1 nmol/L. Among these patients, we saw a deficiency in 173 (82.4%). The people with a normal BMI had an average of 57.6 nmol/L, and the people with a low BMI had the highest average of 60.5 nmol/L. No statistically significant differences were detected between the different BMI categories.

A stratification of the results for the patients aged at least 60 years was then made (Table 4). A slight increase in the level of vitamin D supplementation (10.6%, plus two patients with documented refusal) was noted, which can be interpreted in the context of vitamin D being universally recommended in this age group. With these patients we saw a slight decrease in the level of deficiency in women (73.6%), but an increase in the level of deficiency in men (93.3%).

### 3.3. Albumin Analysis

The albumin levels analysis showed a high level of deficiency in the present study population (Table 3 and Table 4). Using a lower-limit cutoff of 35 g/L, a deficiency was registered in 62.8% of the women and 51.7% of the men. The average level for the men in this study was 34.4 g/L, and for women it was 34 g/L. The average for patients with a normal BMI was 33.1 g/L, and 34.4 g/L for those with a raised BMI. Of the 166 patients with a BMI of 25 kg/m^2^ or higher, 101 were deficient in albumin (60.8%). Again, no statistically significant differences were detected between the different BMI categories. Following a stratification of the patients over 60, we saw a change in the frequency of a deficiency based on sex, with its prevalence in women rising (72%) and a deficiency in men falling (71.1%)

### 3.4. Other Analyses

Somewhat surprisingly, perioperative anaemia was recorded in a number of patients (41.9%), often correlating with a low albumin value (average value 32.6 g/L), possibly indicating malnutrition, aging, or a chronic disease state.

## 4. Discussion

Considering our results, we can see that vitamin D deficiency was a common issue, with a prevalence of 82.1% in the group as a whole. Supplementation was more the exception rather than the rule, with just 18 patients (5.1%) being supplemented. Those under supplementation had, as expected, higher average vitamin D levels (88.9 nmol/L), and deficiency was less common (44.4%). Supplementation was far more common in women than in men (6.4% women and 3.4% of men), and this was also the case for the subgroup of patients aged 60 or older (8.8% of women over 60 and 4.5% of men over 60). Albumin deficiency, while not as prevalent as vitamin D deficiency, was also registered in the majority of patients (58.9%).

The problem of vitamin D deficiency in the general population and its consequences have been well established [10]. Likewise, a systematic review by Iglar et al. in 2015 added evidence to the supposition that surgical outcomes are negatively influenced by low preoperative vitamin D levels in both Orthopaedic and non-Orthopaedic surgical patients [6]. Furthermore, it is important that vitamin D levels are supplemented prior to a procedure and not postoperatively, as postoperative supplementation has not been shown to improve bone healing, at least in cases of fractures [2]. Our study gives concrete evidence about the scale of the problem in the Swiss population. Vitamin D supplementation is recommended for all people in Switzerland aged over 60. In our study population, 170 patients were aged over 60 and just 20 (11.8%) of them had been offered vitamin D supplementation preoperatively (18 accepted, 2 refused).

Our results correlate well with previous analyses of Orthopaedic patients and vitamin D levels. In similar studies conducted on emergency as well as elective Orthopaedic patients in the last 10 years, we have seen levels of sufficiency reaching just 8.7% in India in 2016 and 16% in Germany in 2013 [11,12]. In a study investigating elective Orthopaedic patients in the UK in 2019, sufficiency levels reached 16% [13]. With a total sufficiency level of 17.9% in our study, not much appears to have changed in the last 10 years.

Vitamin D prescribing practices, as well as compliance on the side of patients, have already been investigated in the literature. Uncertainty on the part of physicians as to whether supplementation was necessary or not has been flagged as a major issue. Patients have cited a lack of information as a reason for not adhering to supplementation when it was recommended [14]. According to survey data, the COVID-19 pandemic may have heightened physician awareness of vitamin D supplementation’s benefits [15]. However, comparing our postpandemic study to similar studies conducted before 2019, mentioned above, the deficiency levels are largely unchanged.

One of the major issues may be reimbursement. Health insurance in Switzerland is required to reimburse ambulatory patients for vitamin D levels only in cases of osteomalacia, osteoporosis, fragility fractures, chronic liver/kidney disease, falls without a clear cause, or malabsorption [16]. Outside of these circumstances, patients must carry the cost of vitamin D analysis themselves, even if risk factors like darker skin tones, dietary adjustments, or obesity are present.

Albumin deficiency indicating hypoproteinaemia was, as mentioned above, present in more than half of the patients (58.9%). A low albumin level has been associated with increased mortality in critically ill patients in several studies [17,18]. More recently, adverse outcomes for patients with preoperative low albumin were measured in studies from 2011 and 2014, concerning patients admitted for acute hip fracture [19,20]. Following these results, investigations were made regarding patients admitted for elective Orthopaedic procedures. It has been repeatedly confirmed that patients with low albumin have worse outcomes [21,22,23].

It should be noted that measuring serum albumin alone is not adequate for assessing nutritional status, as albumin levels can remain normal in severely malnourished patients and be reduced in well-nourished patients with acute inflammation, liver problems (synthesis), or proteinuria (insensible loss) [24]. Malnourishment should, in practice, be assessed using a history and clinical exam in combination with blood tests. However, albumin is one of the single most useful blood tests to assess nutritional status, as it has a longer half-life than prealbumin (reflecting long-term nutritional status) and is more readily available and reliable than IGF-1 [25]. Additionally, hypoalbuminemia is a standalone risk factor for postoperative infection and a decreased rate of wound healing [26].

Nutritional screening using a standardized tool (for example, the NRS 2002 criteria) is recommended by the European Society for Clinical Nutrition and Metabolism (ESPEN). It recommends a postponement of a planned operation if the NRS > 5, if weight loss is >10%, if BMI < 18.5 kg/m^2^, or if albumin < 30 g/L [27]. Similar recommendations have been made in the USA by the ASPEN group [28]. Obstacles to preoperative optimisation may include limited access to support from a nutritional therapist or reimbursement, as most oral protein and carbohydrate supplements are not covered by insurers in an outpatient setting.

There have been multiple studies analysing the relationship between bone health and protein intake. Calcium and protein interact constructively to affect bone health. Intakes of both calcium and protein must be adequate to fully realise the benefit of each nutrient for bone [29]. Animal studies have demonstrated that a protein-deficient diet leads to reduced bone mass density and depresses the bone formation rate [30]. Studies in humans have shown that inadequate protein intake can lead to secondary hyperthyroidism within days and can persist for weeks [31]. Finally, in a systematic review, the pooled hazard ratio of all the patients showed that the patients who had a protein intake that was above the recommended daily allowance (RDA) for adults, 0.8 g/kg body weight, had a better bone mineral density (BMD) on average. Although there have been concerns about protein increasing calcinuria, the effect seems to be offset by the increased absorption of calcium from the gastrointestinal tract [32].

Independently of these findings, a strong relationship between serum albumin levels and bone health has been shown. In a study by d’ Erasmo et al. in 1999, a possible link between bone density and low albumin was investigated. They found a statistically significant correlation between low albumin and the development of osteoporosis in patients with a chronic illness (the probable cause of hypoalbuminemia in those patients), but no significant correlation in patients with low albumin and no identifiable cause (i.e., no chronic illness) [33]. The matter was further investigated by means of a cross-sectional observational study by Afshinnia et al. in 2016. In that study, data from over 21,000 outpatients over a period of 12 years were evaluated. An inverse relationship between serum albumin levels and the prevalence of osteoporosis was observed. The association of lower levels of serum albumin with osteoporosis was highly dose responsive, and noted at both the femur neck and lumbar spine, even when confounding variables were accounted for. Interestingly, there was also a measurable association between osteoporosis and the observed duration of hypoalbuminemia [34]. It should also be noted that patients undergoing elective orthopaedic procedures benefit from protein supplementation even if albumin levels are considered to be within a normal range and no concerns about malnutrition are present. In multiple systematic reviews, the benefit from protein supplementation was shown to be conferred during the rehabilitative phase, the primary benefit being decreased muscle atrophy measured by muscle cross-sectional area [35,36].

Our study might have several limitations. We do not have information on other factors driving vitamin D deficiency. The ethnic group, comedications, comorbidities, diet, and information regarding whether the patients were institutionalized or not (care home residents, as well as prisoners, tend to receive less sun exposure than average people do) were not known. The body weight and BMI as absolute values were registered, but no information on NRS scores or recent weight loss was recorded. The albumin level has, therefore, been used as a surrogate for poor nutritional status for the reasons mentioned above. It was also not feasible to measure if the albumin-deficient patients in this cohort had poorer outcomes, which could have added weight to the arguments made here.

Possible solutions to these issues would likely require initiatives addressing both patients and doctors, in particular for vitamin D. A campaign to encourage vitamin D supplementation by patients was run in 2022 by Food Standards Scotland. In an audit following the campaign, awareness of the issue had significantly increased, though predominantly among younger age groups, as the campaign was mostly run on online platforms. Older patients may benefit from print and/or face-to-face channels [37]. There are already campaigns running in Switzerland to try and improve doctors’ sensibility to the usefulness of vitamin D testing and vitamin D supplementation, including “Choosing Wisely Switzerland” [38], and it is conceivable that similar campaigns could also be used to improve the awareness of protein supplementation.

## 5. Conclusions

The need for optimal vitamin D and albumin levels perioperatively has been repeatedly proven in studies. Our study demonstrates that, despite the known need to supplement with vitamin D and have healthy nutrition, low levels of vitamin D and albumin are still very frequent in the Swiss elective Orthopaedic surgery patient population. The adequate workup and treatment of these patients must be continually emphasised if postoperative complications are to be kept to a minimum. The question as to how the awareness of the problem can be improved remains open.

## Figures and Tables

**Table 1:** Demographic data. Abbreviations: BMI, body mass index; ASA, American Society of Anaesthesiologists.

Total Patients	n = 336
Women	n = 218 (64.9%)
Men	n = 118 (35.1%)
Age, mean (range)	59.4 years (17–89 years)
BMI, mean (range)	26.8 (17.8–37.6)
ASA score, mean (range)	II (I–III)

**Table 2:** Perioperative laboratory values. Abbreviations: Lab, laboratory; SD, standard deviation.

	Vitamin D3		Albumin	Haemoglobin	Calcium	Phosphate
	Preoperative Supplementation	Perioperative Insufficiency	Perioperative Insufficiency	Perioperative Deficit	Perioperative Deficit	Perioperative Deficit
Patients	n = 18 (5.3%)	n = 276 (82.1%)	n = 198 (58.9%)	n = 141 (41.9%)	n = 101 (30%)	n = 103 (30.1%)
Average Lab Levels (SD)		56.5 nmol/L (±24.6)	32.7 g/L (±5.6)	122.9 g/L (±13.9)	2.2 mmol/L (±0.3)	0.8 mmol/L (±0.6)

**Table 3:** Results. Abbreviations: SD, standard deviation.

	Vitamin D3		Albumin	
	Women	Men	Women	Men
Deficiency	n = 170 (77.9%)	n = 106 (89.8%)	n = 137 (62.8%)	n = 61 (51.7%)
Normal	n = 48 (22.1%)	n = 12 (10.2%)	n = 20 (9.2%)	n = 57 (48.3%)
Average lab levels (normal and deficient) (SD)	49.1 nmol/L (±15.1)	46.4 nmol/L (±15.6)	32.4 g/L (±2.2)	32.5 g/L (±2.2)
*p*-value between women and men		>0.05		>0.05
Average lab levels overall (normal and deficient) (SD)	57.7 nmol/L (±23.1)	53 nmol/L (±26.5)	34 g/L (±4.2)	34.4 g/L (±5.4)
*p*-value between women and men		>0.05		>0.05

**Table 4:** Results for patients over 60 years of age. Abbreviations: SD, standard deviation.

	Vitamin D3		Albumin	
	Women Age > 60 Years	Men Age > 60 Years	Women Age > 60 Years	Men Age > 60 Years
Deficiency	n = 92 (73.6%)	n = 42 (93.3%)	n = 90 (72%)	n = 32 (71.1%)
Normal	n = 33 (24.4%)	n = 3 (6.7%)	n = 35 (28%)	n = 13 (28.9%)
Average lab levels (normal and deficient) (SD)	56.5 nmol/L (±24.1)	56.1 nmol/L (±24.4)	33 g/L (±4.3)	33 g/L (±2.8)
*p*-value between women and men		>0.05		>0.05
Average lab levels (normal and deficient) (SD)	56.3 nmol/L (±24.4)	56.2 nmol/L (±24.4)	34.1 g/L (±4.3)	34.1 g/L (±4.3)
*p*-value between women and men		>0.05		>0.05

## Data Availability

Datasets can be made available on request to C. Kingston.
